# Penile Mondor’s disease: Clinical and sonographic images

**DOI:** 10.1002/ccr3.2469

**Published:** 2019-10-07

**Authors:** Alain Mwamba Mukendi, Florence Mahlobo

**Affiliations:** ^1^ Department of Urology Chris Hani Baragwanath Academic Hospital University of the Witwatersrand Soweto South Africa; ^2^ Department of Radiology Chris Hani Baragwanath Academic Hospital University of the Witwatersrand Soweto South Africa

**Keywords:** Mondor's disease, penile disorder, superficial dorsal penile, thrombophlebitis

## Abstract

Penile Mondor's disease or thrombophlebitis of the superficial dorsal penile vein is a rare disorder of the penis. Reported cases in the literature are mostly focal thrombus. We present clinical and sonographic images of an extensive superficial dorsal penile vein thrombosis.

## Graphical abstract

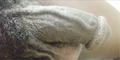

## 1 DESCRIPTION

A 29‐year‐old man presented with a 1‐week history of painful penis that worsens with morning erections. He reported vigorous sexual intercourse 2 days prior to the onset of pains.

**Figure 1:**
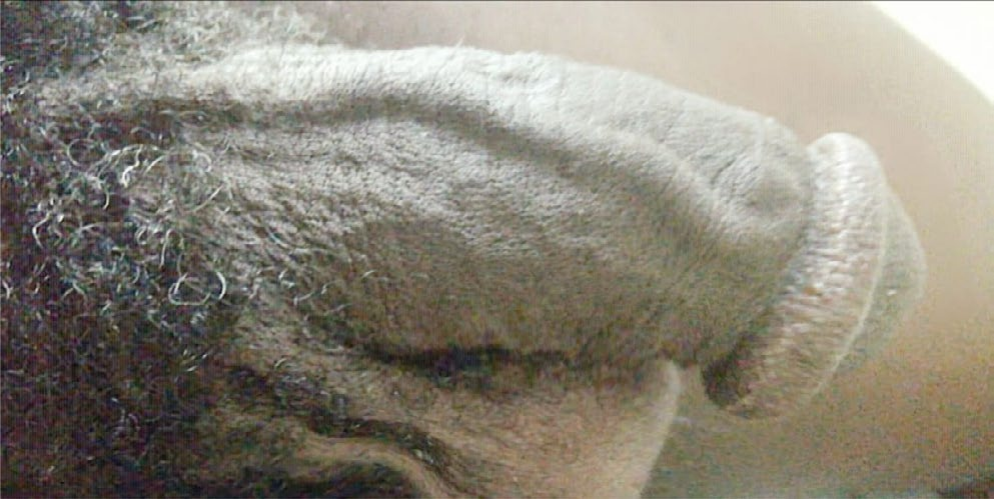
Penile image showing a dilated and tortuous superficial dorsal vein of the penis

**Figure 2:**
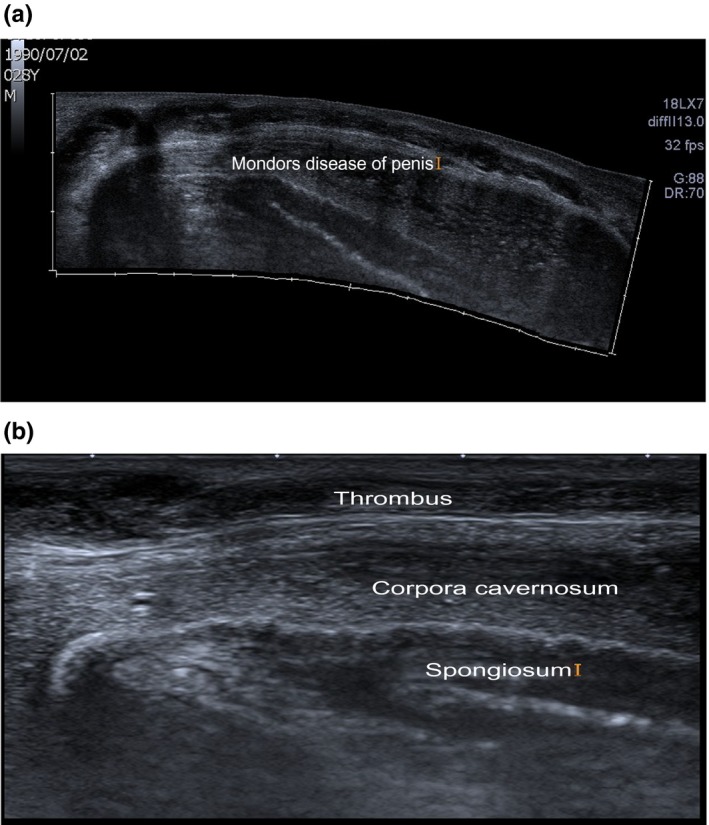
A, Panoramic ultrasound view showing the extensive thrombus from base of the penis to the corona. B, Sonographic image of the penis showing the thrombus, corpora cavernosum, and the spongiosum

Penile examination revealed an 8 cm hard cord‐like and mildly tender structure on the dorsal aspect of the penis extending from the base to the corona (Figure 1). Penile Color Doppler Ultrasound (CDU) demonstrated a thrombosed superficial dorsal penile vein with a thrombus seen from the base of the penis to the junction of the penile shaft and the glans; there was no venous flow detected on Doppler (Figure 2A,B) in keeping with penile mondor's disease (PMD).

Penile mondor's disease is a rare and self‐limiting condition. The most common cause is considered to be trauma due to vigorous and prolonged sexual intercourse.^1^

The condition is self‐limiting within 4‐6 weeks with the vessel regain permeability in 9 weeks. Management is generally medical with sexual activity restriction, anti‐inflammatory medications, and anticoagulation drugs (such as topical heparin).^2^ Our patient was treated with anti‐inflammatory drugs (indomethacin 50 mg per os thrice a day) and topical heparin gel application twice a day. He reported resolution of pains at 2 weeks follow‐up visit before defaulting his next appointment and never came back.

Surgical management is offered to refractory cases to medical treatment defined as persistent symptoms and no venous flow on CDU after 6 weeks of treatment, and it consists of thrombectomy and resection of the superficial penile vein.^2^

## CONFLICT OF INTEREST

None declared.

## AUTHORS CONTRIBUTIONS

AMM: conceived and designed the study, acquired the data, analyzed and interpreted the data, and wrote the manuscript and approved the final manuscript with critical content. FM: acquired, analyzed and interpreted the data.

## References

[1] Singh M , Dalal S , Chhabra T , Bhatia C . Penile Mondor’s disease: a case report and review of literature. Ann Clin Case Rep. 2017;2:1416.

[2] Öztürk H . Penile Mondor’s disease. Basic Clin Androl. 2014;24:5. 25780580 10.1186/2051-4190-24-5 PMC4349227

